# An experiment on individual ‘parochial altruism’ revealing no connection between individual ‘altruism’ and individual ‘parochialism’

**DOI:** 10.3389/fpsyg.2015.01261

**Published:** 2015-08-20

**Authors:** Philip J. Corr, Shaun P. Hargreaves Heap, Charles R. Seger, Kei Tsutsui

**Affiliations:** ^1^Department of Psychology, City University LondonLondon, UK; ^2^Department of Political Economy, King’s College LondonLondon, UK; ^3^School of Psychology, University of East Anglia, NowichUK; ^4^Department of Economics, University of BathBath, UK

**Keywords:** parochial altruism, in-group bias, pro-sociality, personality

## Abstract

Is parochial altruism an attribute of individual behavior? This is the question we address with an experiment. We examine whether the individual pro-sociality that is revealed in the public goods and trust games when interacting with fellow group members helps predict individual parochialism, as measured by the in-group bias (i.e., the difference in these games in pro-sociality when interacting with own group members as compared with members of another group). We find that it is not. An examination of the Big-5 personality predictors of each behavior reinforces this result: they are different. In short, knowing how pro-social individuals are with respect to fellow group members does not help predict their parochialism.

## Introduction

Is parochial altruism an attribute of individual behavior? It is well known from experiments that there is an in-group bias in pro-sociality at a population level. That is, populations reveal that they are nicer to members of their own group than to those of the out-group (e.g., see surveys in Balliet et al., 2014 and Lane, 2015). This is true even for “minimal groups” in which bias is created from the assignment of arbitrary group-memberships (Tajfel, 1970). What is not known is whether the individual variation in the level of pro-sociality (the ‘altruism’) is connected to the individual variation of the in-group bias (the ‘parochialism’). Are those individuals who are most pro-social to insiders also the individuals who are most ‘parochial’ in their pro-sociality? This is the question we address with an experiment^1^.

The question is important because both the social identity and the evolutionary accounts of the emergence of ‘altruism’ would seem to predict such an individual association with ‘parochialism’. For example, the more you identify with your group under social identity and self categorization theory (i.e., the more parochial you are; Turner et al., 1987), the greater should be your anticipated within-group pro-sociality (because this is how you identify your self). Likewise, the Choi and Bowles (2007) agent based simulations predict (under some conditions) the evolutionary emergence of ‘altruism’ but only when combined at the individual level with parochialism.

We address the question in two ways. First, we adopt a revealed preference approach. We examine whether the individual pro-sociality that is revealed in two person public goods (PG) and trust (T) games when interacting with fellow group members helps predict individual parochialism. For this purpose, we measure pro-sociality by the level of ‘contributing’ in the PG and the level of ‘giving’ and ‘returning’ in T; and we measure parochialism by the extent to which individuals’ pro-sociality toward fellow group members does not extend to members of another group (that is, the in-group bias).

The ‘contribution’ rate in PG and the ‘return’ rate in T are commonly taken as an indices of pro-sociality because the selfishly rational individual contributes zero and because a variety of specific non-selfish motivations (like altruism, inequality aversion and a concern for efficiency) predict increasing ‘contribution’ with the strength of these motivations (e.g., see Elster, 2007, on theoretical justifications for this and Camerer, 2003, for a summary of the experimental evidence). The ‘giving’ rate in T is not so easily interpreted because a non-zero gift is consistent with selfishness, when a selfish first mover expects (for whatever reason) that the second mover will ‘return’ a more than compensating amount, as well as with a variety of pro-social motivations like altruism, etc. With this in mind, ‘giving’ in T, is often treated as index, but a noisy one, of pro-sociality. Three possible group contingent measures of pro-sociality follow.

(1) General pro-sociality (i.e., when there are no groups)
(2) In-group pro-sociality (i.e., when both individuals belong to same group)
(3) Out-group pro-sociality (i.e., when the two individuals belong to different groups).

We now define the following for each individual.

• In-group bias = in-group pro-sociality minus out-group pro-sociality.
• In-group ‘love’ = in-group pro-sociality minus general pro-sociality.
• Out-group ‘hate’ = general pro-sociality minus out-group pro-sociality.

The in-group bias is a natural indicator of parochialism because it captures the extent to which insiders are treated differently to outsiders: it is a measure of the extent to which pro-socialitry is restricted to fellow group members. Furthermore, it can conveniently be decomposed with these definitions into in-group ‘love’ plus out-group ‘hate.’

With the background expectation from social identity theory and the Choi and Bowles (2007) evolutionary account, our first approach to the question can now be summarized through H1.

H1: Greater/lesser individual in-group pro-sociality revealed in PG and T is associated, respectively, with greater/lesser individual in-group bias in PG and T.

Second, we complement the revealed preference approach of H1 by considering whether there is a psychological link between altruism and parochialism in the sense that the same personality variables help predict both pro-sociality and the in-group bias. For this purpose, we use the Big-5 personality traits (McCrae and Costa, 1999) as possible predictors. They are ‘Openness,’ ‘Extraversion,’ ‘Agreeablenes,’ ‘Conscientiousness,’ and ‘Neuroticism.’ The five factor personality model is widely used and has been found to help predict pro-sociality in PG (e.g., see Koole et al., 2001; Pothos et al., 2011, and Volk et al., 2012), in T (see Dohmen et al., 2008) and in other games (e.g., Ben-Ner et al., 2004, for the Dictator game). Typically ‘Agreeableness’ is associated with pro-sociality and other traits, less systematically so. We know of no experimental study that has examined whether these traits are associated with in-group bias revealed by individuals.

With the same background expectation, this leads to H2.

H2: The Big 5 personality traits predicting individual pro-sociality in PG and T also predict the individual in-group bias in PG and T.

To our knowledge neither approach to the question of the link between individual ‘altruism’ and individual ‘parochialism’ has been examined experimentally before. There are experiments that have addressed a related but different version of H1. In these experiments, individuals are often first identified as either pro-social or pro-self, then they consider whether the group of pro-social individuals are more likely to engage in acts of actual belligerence than the pro-self group. This evidence, we shall suggest, is mixed and not always easy to interpret in part because the definition of parochialism is slippery. It also does not address the connection at the level of individuals.

For example, Abbink et al. (2012) first classify individuals through their play in a prisoners’ dilemma game as pro-social or (selfish) egoists. The individuals are formed into two groups of four players and then play a Tulloch group conflict game: that is, each individual makes a contribution to a group fund, the size of which relative to the other group fund, influences the likelihood of winning the prize in the group competition. All members of a group have an equal share if their group wins the prize. They find that those who are classified as pro-social in the prisoners’ dilemma game contribute, on average, more in the group competition game than those classified as egoists. In this way, altruism of acting pro-socially in a prisoners’ dilemma game and the parochialism of investing in conflict seem to go together. There is a difficulty, however, with this interpretation of the evidence for parochialism (as potentially distinct from altruism). The group contest game has a free rider dimension. The prize is like a risky PG. Individual contributions have a small effect on the probability of winning the prize and individuals free ride on the contribution of others in this contest. In this context, it is hardly surprising that the pro-socials contribute more to the group contest fund than do the egoists. That is what pro-socials do: they make contributions to PGs when egoists do not^2^.

De Dreu (2010) similarly first classifies individuals via a social value test (that turns on choosing allocations between one’s self and another) into either pro-socials or pro-selves. Once classified, the subjects choose how much to allocate to a within-group fund and a between-group fund. The contributions to both funds generate a PG for the players’ own group. The difference is that a contribution to the between-group fund also lowers the value of the PG for the out-group. Since the latter actually harms the out-group, De Dreu interprets contributions to the within-group and between-group fund as, respectively, in-group love and out-group hate^3^. There is no difference between types in their contributions to the between-group fund but pro-socials as a group contribute more to the within-group fund than pro-selves. Thus, it seems that social value orientation affects in-group love but not out-group hate (in De Dreu’s sense of these terms).

If parochialism is associated with out-group hate because it harms another group (in the same way that Abbink et al., 2012, associate parochialism with investments in contests that harm the interests of the other group), then this means (and contrary to the suggestion in Abbink et al., 2012) parochialism is not connected to social value orientation and in-group love^4^. This, however, is not the interpretation of parochialism that De Dreu et al. (2010) offer in a related experiment, where the term ‘parochialism’ is explicitly used. In this experiment, they associate parochial altruism with in-group love alone. The difficulty with this interpretation is that in-group love in their definition is just what is revealed by contributing to an own group PG and there is no way of judging whether such behavior is parochial because there is no contrasting behavior for what individuals do in the same decision problem when interacting with members of another group. We cannot tell whether their pro-sociality stops at the boundary of their group or not in this experiment: that is, whether it is parochial^5^. It seems that their justification for this interpretation in-group love as parochial turns on an early observation that ‘As in-group love furthers the power and effectiveness of one’s own group vis- à-vis the competing out-group, in-group love is an indirect way of competing with the out-group (De Dreu et al., 2010, p.1408).’ This is perfectly reasonable when groups are indeed in competition with each other. The problem is that in this experiment the groups are not in a competition where this is the case when making contributions to their own group PG.

There are several nested social dilemma experiments where parochialism in our sense of a weakening of pro-sociality when interacting with members of another group and its connection with pro-sociality toward own group members might be examined. In these experiments individuals belong to one of two sub-groups and they have the opportunity to contribute to an own sub-group PG or a collective PG (one that benefits own and the other sub-group members). The contrast between own sub-group PG contributions and contributions to the collective PG could therefore potentially reveal whether pro-sociality weakens beyond the boundary of the own sub-group. The difficulty with interpreting the results of these experiments is that they were not designed for this purpose. Individuals in these experiments have to choose how to allocate their fixed endowment between the two PG accounts and a private one (e.g., see Wit and Kerr, 2002; Polzer, 2004; Halevy et al., 2012). As a result, for any given level of contribution to the private account, there must be a negative association between the contributions to the two PGs accounts. What the relation is between contributions to the two kinds of PGs is not therefore something that is revealed by behavior in these experiments because a negative association is built in by the design of the experiment.

There are four important differences in our experiment. First, by measuring individual pro-sociality by the extent of contributing, giving and returning, we allow for greater granularity in individual pro-sociality than the binary division in these experiments between two types (the pro-social and pro-self). Second, this in turn means that we have individual measures which enable us to examine whether parochialism is associated with pro-sociality at the level of the individual and not just at the level of groups of individuals. Third, we use a natural definition of parochialism (the extent of the in-group bias) that would admit, in principle, Abbink et al.’s (2012) association of parochialism with actual aggression as an extreme case (that is, where pro-sociality has become so weakened as to become negative). It also has the further advantage of being formally connected to our definitions of in-group love and out-group hate: in particular, parochialism can be decomposed into in-group love and out-group hate. Finally, our experiment does not build in any necessary relation between in-group pro-sociality (i.e., ‘altruism’) and the in-group bias (i.e., ‘parochialism’).

## Materials and Methods

Our subjects engage in two counterbalanced tasks. The first task consists of two decision making experiments where pro-sociality has typically been revealed in varying degrees across individuals: the PG and Trust (T) games. The second task is the DeYoung et al. (2007) version of the Big 5 personality survey test. These tasks are counterbalanced to enable control for any possible priming effect of one task upon the other. There are two treatments for the PG and T decisions: one with no group affiliations and the other with minimum, artificial group affiliations where subjects belong to either the red or the blue group. We chose the PG and T games and we used a minimal, artificial group affiliation mechanism because they have revealed the in-group bias in pro-sociality in previous experiments (see Chen and Li, 2009 and Hargreaves Heap and Zizzo, 2009).

In both treatments, subjects anonymously make the PG and T decisions (in a random order) eight times in two separate stages. The decisions are always made with a randomly drawn co-player but in the group treatments the randomness is constrained to ensure equal numbers of interactions with own-group and out-group members. In the T decision, a player occupies the first and second mover roles four times each (i.e., twice in each stage). In the group treatments, subjects know their own color group and that of their co-player; and the random matching is constrained to produce two interactions with co-players from the same group and two from the other group in each of the two stages. At the end of stage 1, a table is shown with the mean contribution rate in the PG and the mean giving and return rates in T. In the group treatment, these values are reported for the following four cases: in-group matching of Blue to Blue and Red to Red, and out-group matching of Blue to Red and Red to Blue. The interactions are split into two stages to allow for possible learning and the introduction of information.

The pay-off details for the PG and T decisions are as follows.

PG: Each player is endowed with 50 experimental points and each must decide how much individual investment to make in the common fund. Individual payoff = 50 – Individual Investment + 0.7 (Total Investment in Common Fund).
T: The first mover is endowed with 50 experimental points and must decide how much (=‘*x*’) “to give” to the second mover. This sum (*x*) is multiplied by three and so the second mover receives 3*x*. The second mover decides how much (=‘*y*’) of 3*x* to return to the first mover. Hence

        First mover payoff = 50 - x + y;
        Second mover payoff = 3x - y.

Our index of individual pro-sociality (for all three group contingent settings) is the amount of ‘contributing’ in PG and ‘giving’ and ‘returning’ in T (with a suitable qualification regarding the possible noisiness in relation to ‘giving’). We express these in terms of % of endowment for ‘contributing’ and ‘giving’ and as % of what becomes the second movers endowment in T (i.e., three times what has been ‘given’ by the first mover).

Our measure of individual parochialism is the in-group bias: the difference in individual pro-sociality when interacting with an insider and outsider. That leaves open the question of whether this should be measured as an absolute number or normalized, say by the level of insider pro-sociality. We normalize because using the absolute value of the gap necessarily builds in a relation between this and individual pro-sociality. To see this, let the mean individual in-group and out-group contributing/giving/returning rate of subject *i*, who participated in the two-group treatment, be *X_i_* and *Y_i_*, respectively. Now suppose that everybody simply treated all outsiders a constant fraction (‘*b*’) less nicely than insiders (i.e., *Y_i_* = *bX_i_*, *b* < 1), then the absolute value of the in-group bias (*X_i_–Y_i_*) would grow with *X_i_.* But this would not reflect any difference in treatment of outsiders relative to insiders (since they are always treated less well by the same fraction ‘*b*’). Normalizing the absolute value of the in-group bias by the level of in-group pro-sociality avoids this false association with in-group pro-sociality.

The model we use in testing H1 is therefore given by

(1) $$\frac{X_{i}-Y_{i}}{X_{i}}={\text{ β}}_{0}+{\text{ β}}_{1}X_{i}+Z_{i}\text{ β}+{\text{ ε}}_{i}\ldots \ldots \ldots \;$$

where *Z_i_* is the vector of dummy variables. For returning rate, *Z_i_* includes the mean points given by out-group coparticipant for the ‘returning’ decision.

Since *X_i_* appears on both sides of the above equation, we obtain the reduced form:

(2) $$Y_{i}=(1-{\text{β}}_{0}-Z_{i}\text{ β})X_{i}-{\text{ β}}_{1}X_{i}^{2}-{\text{β}}_{i}X_{i}\ldots \ldots .\;$$

If there was an association between the in-group bias and pro-sociality, then β_1_ should be positive (i.e., *X_i_^2^* the coefficient on in equation (2) should be negative).

The experiment was conducted at the University of East Anglia^6^. Subjects were recruited through postings on an online participant pool and message board. A total of 110 subjects were involved in eight sessions. Five sessions with 62 subjects in total had no groups to serve as a control group, and the other three sessions with 48 subjects were our experimental group, which had two groups^7^. The instructions, the control questionnaire and the experiment were computerized with zTree (Fischbacher, 2007). There was a show-up fee for the personality survey test and subjects were paid on the basis of a randomly chosen round from each stage in the PG and T games. For this purpose, experimental points were converted at a rate of 4 p per point^8^.

## Results

**Table 1** provides a summary of the results on the average rates of ‘contributing,’ ‘giving,’ and ‘returning’ under different conditions. They reveal typical levels of pro-sociality in the first column when there are no groups (see Camerer, 2003). The in-group bias is apparent in the comparison of the second and third columns where the ‘contributing,’ ‘giving,’ and ‘returning’ between insiders and outsiders, respectively, is set out for the group treatments. The no-group treatment provides the baseline from which to judge the effect of group membership and the respective contributions of in-group love and out-group hate to the in-group bias. **Table 1**, therefore, suggests that the in-group bias in PG arises from in-group love; whereas in T it arises from a mixture of in-group love and out-group hate.

**Table 1:** Summary of results.

		No groups	Insiders	Outsiders
Contributing rate (PG)	Mean	0.256	0.400	0.258


	Somers’ *d*-value		0.328	-0.004
	(*p*-value)		(0.071)	(0.986)
Giving rate (T)	Mean	0.399	0.486	0.337
	Somers’ *d*-value		0.167	-0.137
	(*p*-value)		(0.455)	(0.538)
Returning rate (T)	Mean	0.218	0.224	0.176
	Somers’ *d*-value		0.017	-0.210
	(*p*-value)		(0.909)	(0.313)

Number of subjects		62	48	

*Contributing [giving] rate = the ratio of the contributed [given] points to the endowment of 50 points. Returning rate = the ratio of the returned points to the points the second mover has received. We apply a non-parametric method to test if contributing, giving, and returning rates when matched with insiders or outsiders are larger than these values in the no-group treatment. Each observation is the mean value at the subject level, thus the sample size is 110. Since subjects interact each other as games are repeated, the independent observation is at the session level. Therefore Somers’*d* is employed to use clustering, instead of a Wilcoxon rank-sum test. The *d*-value can be interpreted as follows: the contribution rate to insiders is 32.8% more likely to be higher than the contribution rate in the no-group treatment than *vice versa*. The significance of the *d*-value is reported inside parentheses.*

A non-parametric analysis reports only mild significance of the in-group bias in PG due to few independent observations. The small number of independent observations at this aggregate level arises because the analysis requires two levels of clustering: subjects nested within sessions and the session (as subjects interact with each other as games are repeated). Hence we now test for the significance of these insights by running an individual regression on ‘contributing,’ ‘giving,’ and ‘returning’ rates using three-level models. There are two dummies: one for the group sessions, which is labeled as ‘In-group Matching + Out-group Matching,’ and the other for when the interaction in groups is with outsiders, ‘Out-group Matching’ An in-group bias is picked up by the latter, because controlling with the first dummy for groups sessions, it reveals any difference in behavior toward outsiders. This is Specification A in **Table 2**, where we also control for possible order effects, stage, and round effects and possible reciprocation effects in the ‘return’ equation by including the amount ‘given.’ The regressions reveal an in-group bias: the out-group matching dummy is significant and negative in all equations.

**Table 2:** Individual pro-sociality regressions.

Variable (Game)		Contributing (PG)	Giving (*T*)	Returning (*T*)
Personality survey before PG&T		0.174 (0.291)	0.0880 (0.326)	-0.110 (0.317)
PG Game before trust game		0.343 (0.319)	-0.131 (0.364)	0.387 (0.353)
Session of eight (vs. 14 ≤) Subjects		1.040*** (0.385)	0.852** (0.401)	0.955** (0.418)
Second stage		-0.184*** (0.0179)	-0.142*** (0.0213)	-0.239*** (0.0279)
Round		-0.0699*** (0.00820)	-0.0247** (0.0104)	-0.153*** (0.0135)
Specification A	In-group matching +	0.771** (0.315)	0.679* (0.361)	0.133 (0.348)
	Out-group matching			
	Out-group matching	-0.382*** (0.0267)	-0.374*** (0.0325)	-0.219*** (0.0424)
Specification B	In-group matching	0.771** (0.315)	0.679* (0.361)	0.133 (0.348)
	Out-group matching	0.388 (0.315)	0.305 (0.361)	-0.0858 (0.348)
Given by first Mover				0.0523*** (0.00123)
Constant		1.771*** (0.256)	2.421*** (0.291)	0.875*** (0.282)

Observations		880	440	360

Regressions are three-level mixed-effects poisson model. Since the range of the dependent variables is non-negative integers, we assume poisson distribution instead of *t*-distribution. These are with random intercepts at both the session and the subject-within-session levels since subjects interact each other as games are repeated. For each regression, we tried two models: specifications A and B. The dummy variable In-group Matching is one if the session has two groups and the subject is matched with a member of the same group, otherwise zero. Out-group Matching is one if the session has two groups and the subject is matched with a member of the other group, otherwise zero. In-Group Matching + Out-Group Matching is equal to one in the two-group treatment and zero in the no-group treatment. Significance levels: *p < 0.10, **p < 0.05, and ***p < 0.01.

We also test for whether there is a distinct in-group love/out-group hate origin for this bias. The fact that outsider dummy in Specification A is significantly negative shows that there is a significant difference between the behavior toward insiders and outsiders, but it cannot easily test for whether this comes from either in-group love/out-group hate or some combination of the two. We do this through the regressions in Specification B where we have separate dummies for insider and outsider matching in the group sessions. The only significant coefficient at 95% level is on the insider matching dummy in the ‘contributing’ equation. Hence, there is clear in-group love in PG which could account for the bias. The insider dummy is only weakly significant in the ‘giving’ equation and so it is more likely that some combination of in-group love and out-group hate generates the bias in T.

Result 1: *There is evidence of individual parochialism in the form of in-group bias in PG and T. In-group love could alone account for this in PG but it is more likely to be a combination of in-group love and out-group hate that explains the in-group bias in T.*

Thus we have an experiment where subjects display ‘altruism’ and ‘parochialism,’ we now turn to a test of our two hypotheses concerning the relation between them. Is individual altruism associated with individual parochialism?

**FIGURE 1:**
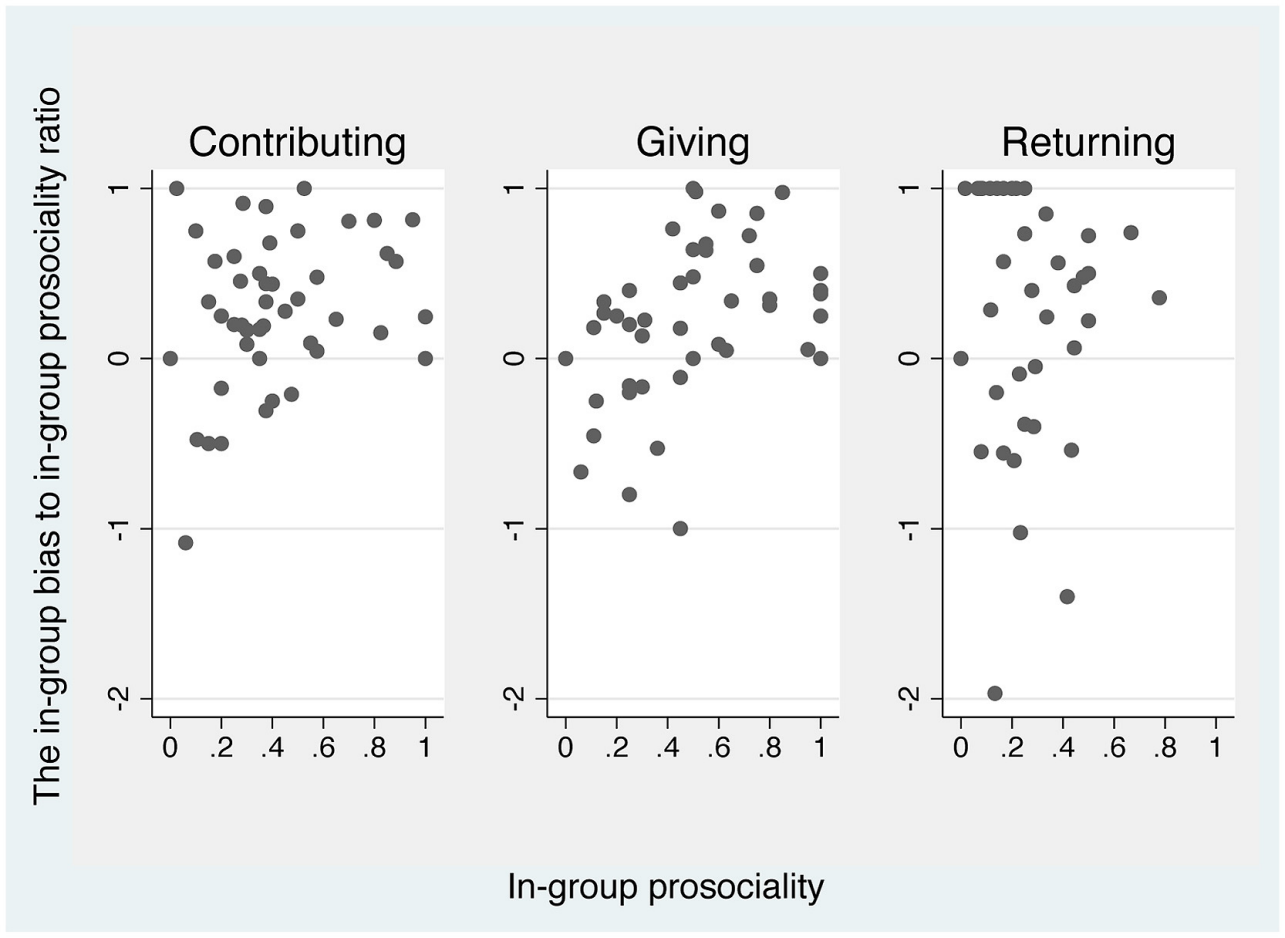
**In-group bias and pro-sociality.** For each subject in the two-group treatment, in-group pro-sociality is derived as the mean in-group contributing/giving/returning rate. The in-group bias is defined as the mean contributing/giving/returning rate of in-group minus that of out-group. When in-group pro-sociality is zero, the observation is omitted except the case in which out-group pro-sociality is also zero. The ratio is set to be zero in this case.

**Figure 1** provides a preliminary view on H1. We plot individual in-group ‘contributing,’ ‘giving,’ and ‘returning’ against the individual in-group bias in each decision when, respectively, normalized by the level of in-group ‘contributing,’ ‘giving,’ and ‘returning.’ The visual evidence is not strong.

We test statistically for this association by estimating equation (2). Due to the potential problem of the heteroskedasticity of the error term, we use the GLS. Recall if there is an association between the normalized in-group bias and pro-sociality, then β_1_ should be positive (i.e., the coefficient on *X_i_^2^* should be negative). This, however, is not what is revealed in **Table 3**. The coefficient on squared insider pro-sociality (*X_i_^2^*) is insignificant in all regressions. The coefficient on *X_i_* is significant and positive, suggesting that our subjects simply contributed/gave/returned to outsiders a fraction of what they do to insiders.

**Table 3:** Insider and outsider pro-sociality.

Variable	Mean out-group contributing rate	Mean out-group giving rate	Mean out-group returning rate
Personality survey before PG&T ×*X_i_*	0.195*** (0.00635)	0.0970*** (0.00207)	0.293*** (0.0516)
PG game before trust game ×*X_i_*	0.0191 (0.0534)	0.231*** (0.00450)	0.350*** (0.0933)
*X_i_*	0.659*** (0.0551)	0.458*** (0.0809)	0.859*** (0.258)
*X_i_*^2^	-0.227 (0.140)	-0.0315 (0.109)	-0.599 (0.391)
Mean out-group given rate ×*X_i_*			-0.590 (0.607)
Observations	48	48	47

Each observation is the mean value at the subject level. Regressions are GLS with clustering at the session level. Significance levels: *p < 0.10, **p < 0.05, and ***p < 0.01.

Result 2 (against H1): *There is no association between normalized individual pro-sociality and individual parochialism in PG and T. There is evidence that individual pro-sociality toward outsiders is a constant fraction of individual pro-sociality toward insiders.*

H2 takes up our second approach to the question of whether individual altruism is connected to individual parochialism. **Table 4** reports on the signs of the significant coefficients when we introduce the Big-5 personality predictors into the regression equations like those in **Table 2** on ‘contributing,’ ‘giving,’ and ‘returning.’ Each personality trait is introduced by itself to capture its general possible influence and in interaction with the two dummies for group sessions and inter-group matching. It is the latter, recall, that captures any influence on the in-group bias. Hence, when the interaction dummy coefficient is negative, this trait contributes to the bias, while a positive coefficient means that the trait counters the bias by promoting pro-sociality toward outsiders. The full regression results are contained in the Appendix, we focus in **Table 4** only on the sign of the personality variables that are significant in predicting pro-sociality in general and the in-group bias.

**Table 4:** Big 5 Predictors of general pro-sociality and in-group bias.

	General		Out-group matching	
	Contributing	Giving	Contributing	Returning
**Big 5**				
Openness			+	
Extraversion			+	-
Conscientiousness			+	-
Agreeableness	+	+	+	
Neuroticism			-	-

‘Agreeableness’ is the only personality trait that (positively) predicts general pro-sociality, i.e., in ‘contributing’ and ‘giving.’ ‘Agreeableness’ also predicts positively pro-sociality with respect to outsiders in ‘contributing’ and ‘returning.’ Thus the one personality predictor of pro-sociality in general works against the in-group bias because it also helps predict pro-sociality with outsiders. There are several traits that have a negative effect in out-group matching (i.e., contribute to the in-group bias) but none helps predict pro-sociality in general.

Result 3 (against H2): *There is no personality trait that helps predict both individual pro-sociality and individual in-group bias in either PG or T.*

## Discussion and Conclusion

The central role of ‘agreeableness’ in predicting pro-sociality is a common finding in the literature where the Big-5 has been used to predict behavior in the PG, T (and Dictator) games (see Koole et al., 2001; Ben-Ner et al., 2004; Dohmen et al., 2008; Pothos et al., 2011; Volk et al., 2012). In this respect, along with the general levels of pro-sociality, our results are consistent with those in the experimental literature. Furthermore, our results tend to support the suggestion in the literature that the in-group bias comes predominantly from in-group love rather than out-group hate (see Balliet et al., 2014). There have been earlier examinations of whether ‘altruism’ is associated at the individual level with ‘parochialism.’ The evidence is not always easy to interpret and is mixed. We are the first, to our knowledge, to examine whether individual pro-sociality is linked to individual parochialism (as captured by the in-group bias) in such detail. This association is important because it is an implication of both social identity and evolutionary accounts of the origins of altruism.

Our measure of pro-sociality is ‘contributing and ‘giving’ and ‘returning’ in the PG and T games, respectively. Our measure of parochialism is the in-group bias in these decisions: the extent to which subjects are less pro-social with outsiders than insiders. We find there is no association. There is an in-group bias, but this does not vary with the level of pro-sociality toward insiders. This result is reinforced by the analysis on the personality predictors of pro-sociality and the in-group bias. ‘Agreeableness’ is the only predictor of pro-sociality and it does not predict its diminution with outsiders (which is what would be expected if altruism was to be linked with parochialism at the individual level). Instead, ‘agreeableness’ positively predicts pro-sociality with both insiders and outsiders. This personality variable, therefore, would lead one to expect that pro-sociality toward insiders moves in tandem with that toward outsider. This is, indeed, what we find. In a complementary result, we find that there are a range of personality predictors of the in-group bias, but none helps predict pro-sociality in general.

Of course, these results are preliminary and need further investigation. One problem is that there are no operational, agreed definitions of the term ‘parochial altruism.’ We have used what we regard as natural definitions, but with different definitions, there may be different results. This suggests the need for further work to clarify how best to define the term. Another problem that future research should address is that our results apply to only two decision settings. It would be good to examine whether the same results hold across further decision problems (e.g., the dictator game, contest games, etc). Nevertheless, if pro-sociality is not tied to parochialism at an individual level, in the way that we have found, then it carries an encouraging implication. An increase in individual ‘altruism’ need not be accompanied by the growth of individual ‘parochialism.’

## Conflict of Interest Statement

The authors declare that the research was conducted in the absence of any commercial or financial relationships that could be construed as a potential conflict of interest.

## Footnotes

1 ‘Altruism’ is sometimes used to capture a very specific attention to the interests of others: the individual’s utility function representation of his or her preferences is a weighted sum of own and the other person’s pay-offs. It is also often used more loosely to describe the motive for behavior that is generally ‘nice’ toward others (that is attends to the interests of the other in one way or another). This could arise from many specific forms of motivation and not just ‘altruism’ in the narrow specific sense of the weighted sum utility function. For example, in many settings behaving ‘nicely’ could arise from a concern with efficiency or equality. We assume here that ‘altruism’ is used in the general rather than specific sense and so stands generally for other regarding behavior (whatever its precise motivation). Hence, we will hereafter typically refer to individual pro-sociality rather than individual ‘altruism’ to avoid any possible confusion.

2 The clean test to avoid this problem would have had individuals deciding whether to enter an individual contest with a member of the other group.

3 This, of course, is related to but is not the same as the definition of in-group love and out-group hate that we will be using. The virtue of our definition is twofold. First, the in-groupness (and out-groupness) of behavior is judged relative to what individuals do when there are no groups, whereas in De Dreu’s (2010) in-group love is simply contributing to an own group PG and there is no way of knowing whether this is special due to a shared group membership because there is no comparison with altruism when there are no groups (see later comments about the difficulties in interpretation that this lack of a comparison can create). Second, the particular point of comparison that we use in our definition has the further benefit of connecting to discussions of social capital in relatively homogenous (where different group ties are weak) as compared with plural societies (where different group ties are strong).

4 This ‘disconnection’ result is also consistent with the closest experiment that we know that considers something like H2. Halevy et al. (2012) where in a similar set up to De Dreu (2010), they find that those who contribute to the within group fund gain ‘prestige’ while those who contribute to the between group fund are regarded as ‘dominant.’ In other words, in-group love and out-group hate involve different psychological currents.

5 This is a natural definition of ‘parochial’ and it admits the Abbink et al. (2012) definition as an extreme version where the weakening is so extreme that hostility actually emerges in relation to other groups.

6 The experiment received approval from the Ethics Committee of the School of Social Work and Psychology at UEA. This committee reports to and acts on behalf of the Social Science Faculty Ethics Committee at UEA. All subjects gave written informed consent at the beginning of the experiment.

7 Two no-group sessions were conducted with eight subjects. One two-group session was conducted with 14 subjects. Other five sessions had 16 subjects.

8 The experiment reported here was part of a larger set of experiments examining discriminatory behavior. The instructions for the full set are contained in the electronic Appendix. Subjects earned on average £16.21 in the experiment that lasted around 90 min and they received a £15 show-up fee for a set of survey questions that lasted between 70 and 90 min.
