# Supplementary material for: An experiment on individual ‘parochial altruism’ revealing no connection between individual ‘altruism’ and individual ‘parochialism’
Source: Front Psychol. 2015 Aug 20;6:1261. doi: 10.3389/fpsyg.2015.01261 (PMC4542132; doi:10.3389/fpsyg.2015.01261)
Supplement: Supplementary file 1 [file Data_Sheet_1.PDF]

Appendix A: Experimental Instructions

A.1 General Instructions

This is an experiment on decision making. Please raise your hand if you have any questions at any point in the experiment.

[Only for Two Group Treatment: There are 16 participants in the experiment, all of which have received the same set of instructions as you have. At the start of the experiment you are randomly assigned to **either the Blue group or the Red group.** Overall, 8 participants are assigned to each group.]

The experiment is divided into **four tasks**. Your final earnings will be equal to the sum of earned points in all tasks. The points are converted into pounds at the rate of 4 pence per experimental point.

A.2 Trust Game Instructions

1. **Introduction**

   Task 1 is divided into two stages. Each stage consists of 4 rounds.

   You will be matched in each round at random with one other participant (the **coparticipant**). [Only for Two Group Treatment: You will be matched **within your group** for **two** rounds out of four, and with coparticipants **from the other group** for the remaining **two** rounds.] As a result of these matching rules, the coparticipant is very likely to change from round to round.

2. **Round Decisions**

   Give/Return Decisions: Each round one of you will be designated to move first. The **First Mover** will begin by receiving 50 points. He or she will decide how many points (if any) to give to the other person and how many (if any) to keep.

   All the points given get multiplied by **3** before they are received by the **Second Mover**. The Second Mover then decides how much (if any) to keep and how much (if any) to return to the First Mover.

   Role: You will have each of the two roles - that of First Mover and that of Second Mover - [One Group Treatment: you will be First Mover for two rounds out of four and Second Mover for the remaining two.][Two Group Treatment: once when matched with a coparticipant within your group and once when matched with a coparticipant from the other group.]

3. **Payments**

   The decisions that you and your coparticipants make will determine the amounts you gain as round payments. Specifically, at the end of the experiment a **winning round** is chosen at random from each stage.

Questionnaire

Please answer the following questions and click the OK button.

1. "If the **First Mover** gives 8 points, the **Second Mover** can return any amount between 0 and 32 points". Is this true? [Yes/No]
2. "If the **First Mover** gives 42 points, the **Second Mover** can return any amount between 0 and 126 points". Is this true? [Yes/No]

Answers

1. "If the **First Mover** gives 8 points, the **Second Mover** can return any amount between 0 and 32 points". Is this true?
   [If answered 'Yes': The correct answer is No.][If answered 'No': That is the correct answer.]
2. "If the **First Mover** gives 42 points, the **Second Mover** can return any amount between 0 and 126 points". Is this true?
   [If answered 'Yes': That is the correct answer.][If answered 'No': The correct answer is Yes.]

[If both questions are answered correctly: Please wait until the experiment continues.][If at least one of the questions are answered incorrectly: Please wait for the experimenter to check your answers.]

Task 2 Public Goods Game Instructions

1. **Introduction**
   Task 2 is divided into two stages. Each stage consists of 4 rounds.
   You will be matched in each round at random with one other participant (the **coparticipant**). [Only for Two Group Treatment: You will be matched **within your group** for **two** rounds out of four, and with coparticipants **from the other group** for the remaining **two** rounds.] As a result of these matching rules, the coparticipant is very likely to change from round to round.
2. **Round Decisions**
   Each round you and your coparticipant are given 50 points each. Your decision is whether to invest any amount from 0 to 50 points in a Common Fund. It is entirely up to you whether to invest or not, and you keep for yourself any amount which you choose not to invest. For each point that you and your coparticipant invest, you both receive 0.7 points for the total amount invested. In general, you will receive:
   Payment = 50 – Your Investment + 0.7 (Total Investment in Common Fund)
   Example: *If you invested 10 points and your co-participant invested 40 points (these numbers are purely illustrative), the total amount invested is 50 points and the return to each of you is 70% of this, i.e. 35 points. For this round, therefore, you will have 50 – 10 + 35 =75 points and your co-participant will have 50 – 40 + 35 = 45 points as the final earnings for the round.*
   After both you and your coparticipant have made your decisions, you will be told how much each of you invested and how much each of you earned.
3. **Payments**
   The decisions that you and your coparticipants make will determine the amounts you gain as round payments. Specifically, at the end of the experiment a **winning round** is chosen at random from each stage.

Questionnaire
Please answer the following questions and click the OK button. You can see the instructions again by clicking the Instructions button.
Some facts you may wish to use in answering this questionnaire:

- 50 – 30 = 20; 30 + 30 = 60.
- 70% of 30 is 21; 70% of 60 is 42.

1. How much does your coparticipant earn if you both invest 0?
2. How much does your coparticipant earn if you both invest 30?
3. How much do you earn if you invest 0 and your coparticipant invests 30?
4. How much do you earn if you invest 30 and your coparticipant invests 0?

Answers

1. How much does your coparticipant earn if you both invest 0?
   [If answered incorrectly: The correct answer is 50 – 0 + 0 = 50.]
2. How much does your coparticipant earn if you both invest 30?
   [If answered incorrectly: The correct answer is 50 – 30 + 42 = 62.]
3. How much do you earn if you invest 0 and your coparticipant invests 30?
   [If answered incorrectly: The correct answer is 50 – 0 + 21 = 71.]
4. How much do you earn if you invest 30 and your coparticipant invests 0?
   [If answered incorrectly: The correct answer is 50 – 30 + 21 = 41.]

A.3 Real-Effort Competition Task Instructions

1. **Introduction**
   Task 3 is divided into two stages. Each stage consists of 2 rounds.
   You will be matched in each round at random with one other participant (the **coparticipant**) [Only for Two Group Treatment: You will be matched **within your group** for **one** round out of two, and with coparticipants **from the other group** for the remaining **one** round.] As a result of these matching rules, the coparticipant is very likely to change from round to round.
   You will be asked to subtract a certain number repeatedly, starting from a given number.
   <u>Example:</u>
   Please subtract 2 repeatedly from 20.
   You will solve:
   20 – 2 = 18.
   You will write down in the box at the bottom of the screen 18 and you will submit your answer by cliking the "Submit" button.
   You will see a new screen starting from 18.
   <u>Example:</u>
   You will solve:
   18 – 2 = 16.

You will write down in the box at the bottom of the screen 16 and you will submit your answer by cliking the “Submit” button.
You will see a new screen starting from 16.
<u>Example:</u>
You will solve:
16 – 2 = 14.
You will write down in the box at the bottom of the screen 14 and you will submit your answer by cliking the “Submit” button.
You will see a new screen starting from 14.
You are not allowed to use any type of calculator.
Task 3 will last for 2 minutes. Try to count backwards as quickly as possible within the 2 minutes. The remaining time is displayed in seconds in the top right corner of the screen.
Please click the OK button to move to the next page.

2. **Payments**
The number of correct subtraction that tou and your coparticipants make will determine the amounts you gain as round payments. Specifically, at the ned of the experiment a **winning round** is chosen at random from each stage.
3. **Decisions**
After being matched with a coparticipant, but before knowing the value of correct subtraction and the beginning value, you choose between two options of payment, option A and B.

You choose	and your coparticipant chooses	You personally earn
Option A	Option A or B	**6 points** for each correct answer you have calculated whatever the number of correct answers calculated by your coparticipant.
Option B	Option A	**10 points** for each correct answer you have calculated whatever the number of correct answers calculated by your coparticipant
Option B	Option B	**10 points** for each correct answer you have calculated if you calculated more correct than your coparticipant.<br>**2 points** for each correct answer you have calculated if you answered less correct than your co-participant.<br>In each of a tie, a random draw determines who receives **10 points** and who receives **2 points**

		per correct answer.

Questionnaire

Please answer the following questions and click the OK button. You can see the table of payment rule again by clicking the Table button.

Suppose you correctly anwer **4** times and your coparticipant correctly answer **5** times.

1. How many points for each correct answer do you earn if both you and your coparticipant have selected Optoin A?
2. How many points for each correct answer do you earn if both you and your coparticipant have selected Optoin B?

Suppose you correctly answer **5** times and your coparticipant correctly answer **4** times.

3. How many points for each correct answer do you earn if you have selected Option A and your coparticipant has selected Optoin B?
4. How many points for each correct answer do you earn if you have selected Option B and your coparticipant has selected Optoin A?

Answers

Suppose you correctly anwer **4** times and your coparticipant correctly answer **5** times.

1. How many points for each correct answer do you earn if both you and your coparticipant have selected Optoin A?
   [If answered 6: That is the correct answer.][Else: The correct answer is 6 points.]
2. How many points for each correct answer do you earn if both you and your coparticipant have selected Optoin B?

Suppose you correctly answer **5** times and your coparticipant correctly answer **4** times.

3. How many points for each correct answer do you earn if you have selected Option A and your coparticipant has selected Optoin B?
4. How many points for each correct answer do you earn if you have selected Option B and your coparticipant has selected Optoin A?

A.4 Holt and Laury Type Individual Task Instructions

Task 4 is an individual task.

You now need to make 10 decisions for each of three successive computer screens. Each decision is a paired choice between two options (for example, "Option A" and "Option B"). You will make ten decisions and record these in the

final column, but only one of them from each computer screen will be used in the end to determine your earnings. You will only know which one at the end of the experiment.
Before you start making your ten choices, please let us explain how these decisions will affect your earnings for this part of the experiment. After you have made all of your decisions, the computer will randomly select which of the ten decisions will be used to determine your earnings. In relation to this decision, the computer will then randomly select the outcome based on the probabilities assigned to the option you chose.
As an example, assume that, for the randomly selected decision, the option to the left pays 100 points with a 10% chance and 50 points with a 90% chance, while the option to the right pays 80 points with a 20% chance and 45 points with a 80% chance. Assume that you chose the option to the left for this decision; then there is a 10% chance that will earn 100 points and a 90% chance that you will earn 50 points.
Please raise your hand if you have any questions.

Example	100 points with 10% chance<br>50 points with 90% chance	80 points with 20% chance<br>45 points with 80% chacne

Appendix B: Holt and Laury Type Individual Task

Table B1: Gain Task

	Option A	Option B	Your Choice A or B
Decision 1	45 points with 10% chance<br>35 points with 90% chance	85 points with 10% chance<br>2 points with 90% chance	
Decision 2	45 points with 20% chance<br>35 points with 80% chance	85 points with 20% chance<br>2 points with 80% chance	
Decision 3	45 points with 30% chance<br>35 points with 70% chance	85 points with 30% chance<br>2 points with 70% chance	
Decision 4	45 points with 40% chance<br>35 points with 60% chance	85 points with 40% chance<br>2 points with 60% chance	
Decision 5	45 points with 50% chance<br>35 points with 50% chance	85 points with 50% chance<br>2 points with 50% chance	
Decision 6	45 points with 60% chance<br>35 points with 40% chance	85 points with 60% chance<br>2 points with 40% chance	
Decision 7	45 points with 70% chance<br>35 points with 30% chance	85 points with 70% chance<br>2 points with 30% chance	
Decision 8	45 points with 80% chance<br>35 points with 20% chance	85 points with 80% chance<br>2 points with 20% chance	
Decision 9	45 points with 90% chance<br>35 points with 10% chance	85 points with 90% chance<br>2 points with 10% chance	
Decision 10	200 with 100%	385 with 100%	

Table B2: Loss Task

	Option C	Option D	Your Choice C or D
Decision 1	-45 points with 10% chance<br>-35 points with 90% chance	-85 points with 10% chance<br>-2 points with 90% chance	
Decision 2	-45 points with 20% chance<br>-35 points with 80% chance	-85 points with 20% chance<br>-2 points with 80% chance	
Decision 3	-45 points with 30% chance<br>-35 points with 70% chance	-85 points with 30% chance<br>-2 points with 70% chance	
Decision 4	-45 points with 40% chance<br>-35 points with 60% chance	-85 points with 40% chance<br>-2 points with 60% chance	
Decision 5	-45 points with 50% chance<br>-35 points with 50% chance	-85 points with 50% chance<br>-2 points with 50% chance	
Decision 6	-45 points with 60% chance<br>-35 points with 40% chance	-85 points with 60% chance<br>-2 points with 40% chance	
Decision 7	-45 points with 70% chance<br>-35 points with 30% chance	-85 points with 70% chance<br>-2 points with 30% chance	
Decision 8	-45 points with 80% chance<br>-35 points with 20% chance	-85 points with 80% chance<br>-2 points with 20% chance	
Decision 9	-45 points with 90% chance<br>-35 points with 10% chance	-85 points with 90% chance<br>-2 points with 10% chance	

Decision 10	-45 points with 100% chance	-85 points with 100% chance	
You will lose points in this period.			

Table B3: Ambiguity Task

	**Option E**	**Option F**	**Your Choice E or F**
Decision 1	55 points with ?% chance<br>55 points with ?% chance	50 points with 50% chance<br>50 points with 50% chance	
Decision 2	50 points with ?% chance<br>60 points with ?% chance	45 points with 50% chance<br>55 points with 50% chance	
Decision 3	45 points with ?% chance<br>65 points with ?% chance	40 points with 50% chance<br>60 points with 50% chance	
Decision 4	40 points with ?% chance<br>70 points with ?% chance	35 points with 50% chance<br>65 points with 50% chance	
Decision 5	35 points with ?% chance<br>75 points with ?% chance	30 points with 50% chance<br>70 points with 50% chance	
Decision 6	30 points with ?% chance<br>80 points with ?% chance	25 points with 50% chance<br>75 points with 50% chance	
Decision 7	25 points with ?% chance<br>85 points with ?% chance	20 points with 50% chance<br>80 points with 50% chance	
Decision 8	20 points with ?% chance<br>90 points with ?% chance	15 points with 50% chance<br>85 points with 50% chance	
Decision 9	15 points with ?% chance<br>95 points with ?% chance	10 points with 50% chance<br>90 points with 50% chance	
Decision 10	10 points with ?% chance<br>100 points with ?% chance	5 points with 50% chance<br>95 points with 50% chance	
?% chance means that you do not know how likely each outcome is. Two unknown probabilities add up to 100%.			
