# Supplementary material for: An experiment on individual ‘parochial altruism’ revealing no connection between individual ‘altruism’ and individual ‘parochialism’
Source: Front Psychol. 2015 Aug 20;6:1261. doi: 10.3389/fpsyg.2015.01261 (PMC4542132; doi:10.3389/fpsyg.2015.01261)
Supplement: Supplementary file 2 [file Data_Sheet_2.DOCX]

**Table A1: Big 5**

| Variable | Contributing | Giving | Returning |
| --- | --- | --- | --- |
| (Game) | (PG) | (T) | (T) |
| Personality Survey Before PG&T | 0.346 | 0.321 | 0.134 |
|  | (0.292) | (0.309) | (0.320) |
| PG Game Before Trust Game | 0.348 | -0.224 | 0.330 |
|  | (0.312) | (0.338) | (0.350) |
| Session of 8 (vs. 14≤) Subjects | 0.950** | 0.702* | 0.877** |
|  | (0.378) | (0.374) | (0.413) |
| 2^nd^ Stage | -0.187*** | -0.143*** | -0.232*** |
|  | (0.0179) | (0.0213) | (0.0280) |
| Round | -0.0714*** | -0.0209** | -0.147*** |
|  | (0.00825) | (0.0105) | (0.0138) |
| Session of 2 Groups | -1.174 | -4.438 | -4.799 |
|  | (3.929) | (3.101) | (4.375) |
| Out-group Matching | -5.598*** | -0.0510 | 3.986*** |
|  | (0.493) | (0.627) | (0.796) |
| Given by 1^st^ Mover |  |  | 0.0526*** |
|  |  |  | (0.00131) |
| Openness | -0.527 | -0.231 | -0.437 |
|  | (0.348) | (0.278) | (0.389) |
| 2 Groups * Openness | 0.871 | 0.838* | 1.008 |
|  | (0.540) | (0.440) | (0.629) |
| Out-g. match * Openness | 0.198*** | -0.0436 | -0.124 |
|  | (0.0572) | (0.0806) | (0.121) |
| Extraversion | -0.160 | -0.132 | -0.393 |
|  | (0.296) | (0.232) | (0.320) |
| 2 Groups * Extraversion | 0.00554 | 0.0353 | -0.247 |
|  | (0.482) | (0.378) | (0.545) |
| Out-g. match * Extraversion | 0.265*** | 0.00728 | -0.364*** |
|  | (0.0517) | (0.0678) | (0.112) |
| Conscientiousness | -0.105 | -0.353 | -0.310 |
|  | (0.345) | (0.272) | (0.376) |
| 2 Groups * Conscientiousness | 0.108 | 0.375 | 0.270 |
|  | (0.470) | (0.370) | (0.522) |
| Out-g. match * Conscientiousness | 0.163*** | -0.111* | -0.242*** |
|  | (0.0469) | (0.0594) | (0.0713) |
| Agreeableness | 0.957*** | 0.567** | 0.753* |
|  | (0.362) | (0.288) | (0.404) |
| 2 Groups * Agreeableness | -0.717 | -0.142 | 0.0586 |
|  | (0.571) | (0.452) | (0.646) |
| Out-g. match * Agreeableness | 0.469*** | 0.0258 | -0.153 |
|  | (0.0704) | (0.0883) | (0.121) |
| Neuroticism | -0.114 | -0.226 | -0.264 |
|  | (0.242) | (0.194) | (0.264) |
| 2 Groups * Neuroticism | 0.414 | 0.418 | 0.324 |
|  | (0.394) | (0.315) | (0.430) |
| Out-g. match * Neuroticism | 0.360*** | 0.0219 | -0.361*** |
|  | (0.0478) | (0.0604) | (0.0759) |
| Constant | 1.051 | 3.251* | 2.486 |
|  | (2.241) | (1.760) | (2.491) |
| Observations | 864 | 432 | 355 |

*Notes*: See to notes of Table 2 for the model specification.
